# A novel nonsense mutation in *CRYGC* is associated with autosomal dominant congenital nuclear cataracts and microcornea

**Published:** 2009-02-06

**Authors:** Lu Zhang, Songbin Fu, Yangshan Ou, Tingting Zhao, Yunjuan Su, Ping Liu

**Affiliations:** 1 Eye hospital, the First Affiliated Hospital, Harbin Medical University, Harbin, China; 2 Laboratory of Medical Genetics, Harbin Medical University, Harbin, China

## Abstract

**Purpose:**

To report the identification of a novel nonsense mutation in *CRYGC* in a Chinese family with autosomal dominant congenital nuclear cataracts and microcornea.

**Methods:**

We investigated a four-generation Chinese family with six members affected with nuclear cataracts and microcornea. The family resides in a relatively isolated region of northern China. Genomic DNA was isolated from blood leucocytes, genotyping was performed using more than 100 microsatellite markers for the known cataract candidate gene loci, and LOD scores were calculated using the LINKAGE programs. Mutations were detected by DNA sequence analysis of the candidate genes.

**Results:**

Evidence for linkage was detected at marker D2S325 (LOD score [Z]=2.29, recombination fraction [θ]=0.0), which closely flanks the γ-crystallin gene cluster (*CRYGA*-*CRYGD*) on chromosome 2q32.3-q35. Direct sequencing of the candidate *CRYGA*-*CRYGD* gene cluster revealed a c.470G>A transversion in exon 3 of *CRYGC*, which cosegregated with cataracts in the family and was not observed in 100 normal controls. This single nucleotide change was predicted to introduce a translation stop codon at tryptophan 157 (W157X).

**Conclusions:**

The present study has identified a novel nonsense mutation in *CRYGC* associated with autosomal dominant cataracts and microcornea in a Chinese family. Our finding expands the spectrum of *CRYGC* mutations associated with congenital cataract and confirms the role of γ-crystallin in the pathogenesis of congenital nuclear cataracts.

## Introduction

Congenital cataract is a clinically and genetically heterogeneous lens disorder that typically appears as a sight-threatening trait in childhood and accounts for one-tenth of the cases of childhood blindness [1]. The prevalence of congenital cataracts is estimated to vary from 0.6 to 6 per 10,000 live births with an incidence of 2.2-2.49 per 10,000 live births [2]. Approximately half of all congenital cataract cases are inherited either in isolation or as part of a syndrome of ocular or systemic abnormalities [3]. All three classical forms of Mendelian inheritance have been associated with non-syndromic cataracts. However, the majority of families with a history of congenital cataracts show an autosomal dominant transmission pattern.

There are currently 34 loci implicated in nonsyndromic congenital cataract, and of these, over 20 have been associated with mutations in specific genes [4]. These genes can be considered in five functional groups: (1) 10 genes that encode crystallines, *CRYAA* [5,6], *CRYAB* [7,8], *CRYBB1* [9,10], *CRYBB2* [11-14], *CRYBB3* [15], *CRYBA1/A3* [16,17], *CRYBA4* [18], *CRYGC* [19-23], *CRYGD* [19,21,24-26], and *CRYGS* [27]; (2) two genes that encode the gap junction proteins, *GJA8* [28,29], and *GJA3* [30,31]; (3) two genes that encode membrane proteins, *MIP* [32] and *LIM2* [33,34]; (4) two genes that encode beaded filament proteins, *BFSP1* [35] and *BFSP2* [36-38]; and (5) seven genes that encode growth and transcription factors, *PITX3* [39], *MAF* [40,41], *HSF4* [42], *FOXE3* [43], *EYA1* [44], *CHX10* [45], and *PAX6* [46].

Crystallins are the dominant structural components of the vertebrate eye lens. At least 11 crystallin genes encode over 95% of the water-soluble structural proteins present in the crystallin lens, representing more than 30% of its mass and accounting for its optical transparency and high refractive index [47]. Therefore, mutations in the crystallin genes are strong candidates for certain hereditary forms of congenital cataracts.

In this study, we performed linkage analysis of a four-generation Chinese family with autosomal dominant nuclear cataract and microcornea, and we identified a novel nonsense mutation in *CRYGC* on 2q.

## Methods

### Clinical evaluation and DNA specimens

**Figure 1:**
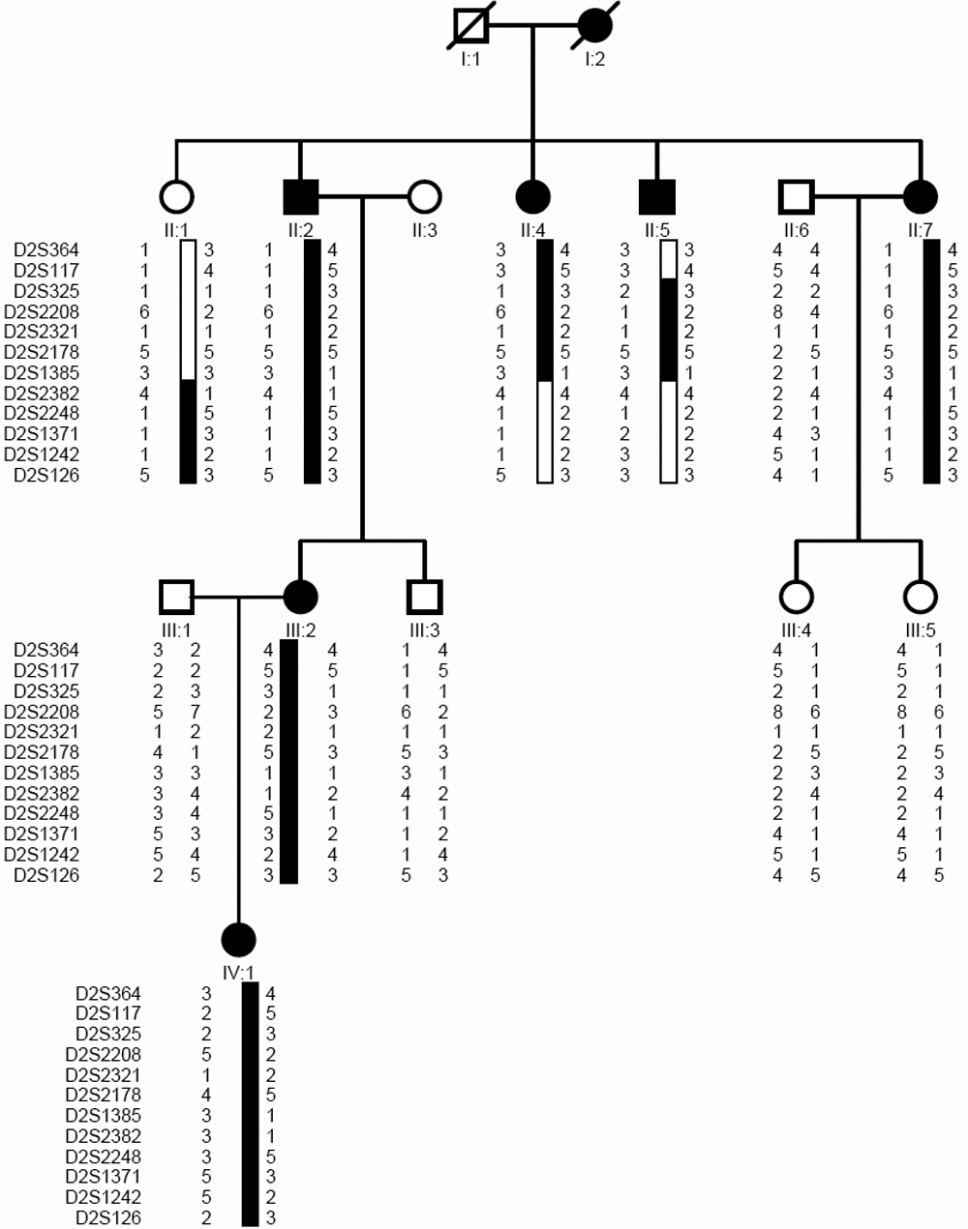
Pedigree and haplotypes of the Chinese family with cataracts. A pedigree is shown with the haplotype analysis of the Chinese family with cataracts, which shows the segregation of 10 microsatellite markers on chromosome 2q in descending order from the centromere. Squares and circles represent males and females, respectively. Black and white symbols denote affected and unaffected individuals, respectively.

We identified a four-generation Chinese family with autosomal dominant nuclear cataracts and microcornea in the absence of other ocular or systemic abnormalities. The family resides in a relatively isolated region of northern China. Informed consent in accordance with the Declaration of Helsinki was obtained from all participants. Thirteen members participated in the study, six affected and seven unaffected family members (Figure 1). Affected status was determined by a history of cataract extraction or ophthalmologic examination, which included visual acuity testing, slit lamp examination, intraocular pressure measurement, and fundus examination with dilated pupils. Phenotypes were documented using slit lamp photography. Peripheral blood was collected and genomic DNA was extracted from blood leukocytes using a QIAampDNA Blood Mini Kit (Qiagen, Hilden, Germany).

### Genotyping and linkage analysis

We conducted a genome wide linkage scan based on a set of dinucleotide repeat microsatellite markers spaced at approximately 10 cM intervals using an ABI PRISM Linkage Mapping Set version 2.5 (Applied Biosystems, Foster City, CA). “Touchdown” polymerase chain reaction (PCR) was performed in a 5 μl reaction volume containing 20 ng of genomic DNA, 1 µl of 10X PCR buffer, 7 mM MgCl_2_, 0.2 mM dNTPs, 0.3 U HotStar Taq DNA polymerase, and 0.05 µM microsatellite markers. After an initial denaturation period of 12 min at 95 °C, 14 cycles were performed at 95 °C for 30 s, 63–56 °C for 30 s (with a 0.5 °C decrease at each step), and 72 °C for 1 min. Thirty cycles were performed at 95 °C for 30 s, 56 °C for 30 s, and 72 °C for 1 min followed by an extension at 72 °C for 10 min and a final hold at 4 °C. The PCR products were pooled on the basis of size (Genescan-400HD ROX; Perkin Elmer, Foster City, CA), denatured at 95 °C for 1 min, and electrophoresed in a 96 capillary automated DNA analysis system (MegaBACE 1000, Amersham, Freiburg, Germany). The results were analyzed by Genetic Profiler version 1.5 (Amersham). Two point LOD scores (Z) were calculated using the LINKAGE software package (version 5.1). A gene frequency of 0.0001 and a penetrance of 100% were assumed for the cataract locus. LOD scores were calculated at recombination fractions (θ) of 0.00, 0.05, 0.1, 0.2, 0.3, and 0.4.

### Mutational analyses

Four functional candidate genes (*CRYGA*-*CRYGD*) and two pseudogenes (*CRYGE*-*CRYGF*) with in-frame translation stop codons are clustered within the physical region defined for the cataract on 2q33–35. We systematically sequenced all four functional *CRYG* genes, each comprising three exons and two introns, in two affected and two unaffected members of the family using specific primers (Table 1). Genomic DNA was PCR amplified, purified, and sequenced directly using dye-terminator chemistry. The purified PCR products were sequenced on both DNA strands using an ABI 3100 sequencer (Applied Biosystems). After identifying a nonsense mutation in exon 3 of *CRYGC*, all of the family members (six affected and seven unaffected family members) and 100 unrelated normal individuals were screened.

**Table 1:** PCR primers for mutational screening of *CRYGA*-*CRYGD*.

**Gene (Exon)**	**Strand**	**Primer Sequence (5′ - 3′)**
*CRYGA* (1–2)	Sense	CCAGGTCCCTTTTGTGTTGT
	Antisense	GGGTCAGGCCTTGCTATTCT
*CRYGA* (3)	Sense	GGCAACACAGCAAGACCTTT
	Antisense	AGCCACTTAGTGCAGGGAAC
*CRYGB* (1–2)	Sense	GCCCTTTTGTGTGATTTCCT
	Antisense	CGAGACTCCGCCTCAAAA
*CRYGB* (3)	Sense	AAACTTGGCCTGGGAGAACT
	Antisense	TTGGCTGAGTGCCATTATCA
*CRYGC* (1)	Sense	GGACAGCGTTAGAATATACCAGAGA
	Antisense	CTGAAATACGGCTGCAGGTT
*CRYGC*(2)	Sense	GGAAGGTGAGCAGAACACAA
	Antisense	TCCATCTAACCCTTAGGTGTTTTT
*CRYGC* (3)	Sense	CATGCCACAACCTACCAAGTT
	Antisense	TGACAAGGAGCATTTAAAGGTG
*CRYGD* (1)	Sense	CAGCAGCCCTCCTGCTAT
	Antisense	TGCTCATAGAGCATCCAGCA
*CRYGD* (2)	Sense	GCCTTGCAGATCACCCTCTA
	Antisense	TAGGGCAGGAGACACATTCC
*CRYGD* (3)	Sense	GCTTGAGCGGGTCCTCAC
	Antisense	GCCTCGTGTGTGTAAATAAAATAAGA

Primer pairs used for amplification and sequencing of the coding exons for *CRYGA-CRYGD* located on 2q.

## Results

### Clinical findings

**Figure 2:**
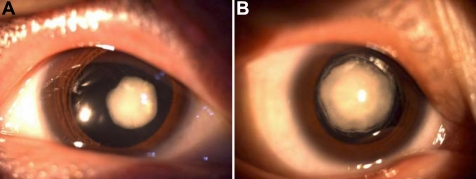
Slit lamp photographs of the eyes of affected individuals. **A**: Individual II:2 at 46 years of age is shown with visual acuity of hand movement in both eyes before surgery. **B**: Individual III:1 at 26 years of age is also shown with visual acuity of hand movement in both eyes before surgery.

We report the identification of a four-generation Chinese family with a clear diagnosis of congenital nuclear cataracts and microcornea. Autosomal dominant inheritance of the cataract phenotype was supported by the presence of affected individuals in each of the four generations and male-to-male transmission. The affected individuals presented with bilateral congenital nuclear cataracts that consisted of a central nuclear opacity affecting the embryonic, fetal, and infantile nucleus of the lens (Figure 2) and with congenital small eyeballs and corneas with a diameter of approximately 9–10 mm. Every affected individual had the same poor visual acuity of hand movement in front of the eye, and they also had nystagmus and amblyopia.

### Linkage analysis

By following the exclusion of large chromosomal regions, we obtained suggestive evidence of linkage for marker D2S2321 (Z=1.88, θ=0) on 2q33.3. Further analysis of the marker showed a positive LOD score at the recombination fraction of 0.00 and strongly supported this candidate region. The maximum two-point LOD score (Z_max_) of 2.29 was obtained at marker D2S325 with recombination θ=0.00. The adjacent markers (D2S2321, D2S1385, and D2S1242) also showed LOD scores greater than 1.0. The results of the two-point LOD scores are summarized (Table 2).

**Table 2:** Two-point LOD scores for linkage analyses.

**cM**	**Markers**	**LOD score at θ=**							
		**0.00**	**0.05**	**0.10**	**0.20**	**0.30**	**0.40**	**Z_max_**	**θ_max_**
186.21	D2S364	−1.84	0.52	0.64	0.55	0.33	0.09	0.64	0.1
194.45	D2S117	−1.84	0.52	0.64	0.55	0.33	0.09	0.64	0.1
204.53	D2S325	2.29	2.07	1.84	1.34	0.79	0.26	2.29	0.0
205.06	D2S2208	0.80	0.79	0.73	0.55	0.33	0.12	0.80	0.0
205.06	D2S2321	1.88	1.69	1.48	1.05	0.58	0.16	1.88	0.0
205.59	D2S2178	0.26	0.31	0.32	0.28	0.19	0.09	0.32	0.1
206.74	D2S1385	1.88	1.69	1.49	1.05	0.58	0.16	1.88	0.0
213.49	D2S2382	−2.01	−0.43	0.01	0.27	0.26	0.13	0.27	0.2
214.71	D2S2248	−2.01	−0.44	−0.00	0.26	0.24	0.12	0.26	0.2
215.25	D2S1371	−1.98	0.55	0.67	0.60	0.39	0.15	0.67	0.1
218.45	D2S1242	1.08	1.05	0.97	0.74	0.45	0.17	1.08	0.0
221.13	D2S126	0.88	0.81	0.72	0.51	0.27	0.07	0.88	0.0

Two point LOD scores for linkage between the cataract locus and twelve markers on chromosome 2q listed in genetic (sex-averaged) order using the Marshfield genetic database from p-tel, measured in centi-Morgans (cM).

We constructed haplotypes of the family. The markers used are listed in Table 2. Haplotype data are given in Figure 1. A crossover between D2S117 and D2S325 in individual II:5 defines the proximal border of the region, and one between D2S1385 and D2S2382 in individual II:1, II:4, and II:5 define the distal border. All affected individuals had an affected parent, and none of the unaffected individuals carried the disease haplotype. Thus, penetrance appears to be virtually complete in this family. The disease-associated haplotype shared by all affected members was identified. The results of both linkage and haplotype analyses situated the disease gene in a 19.04 cM region bounded by D2S117 and D2S2382 at 2q32.3-q35.

### Mutational analysis

**Figure 3:**
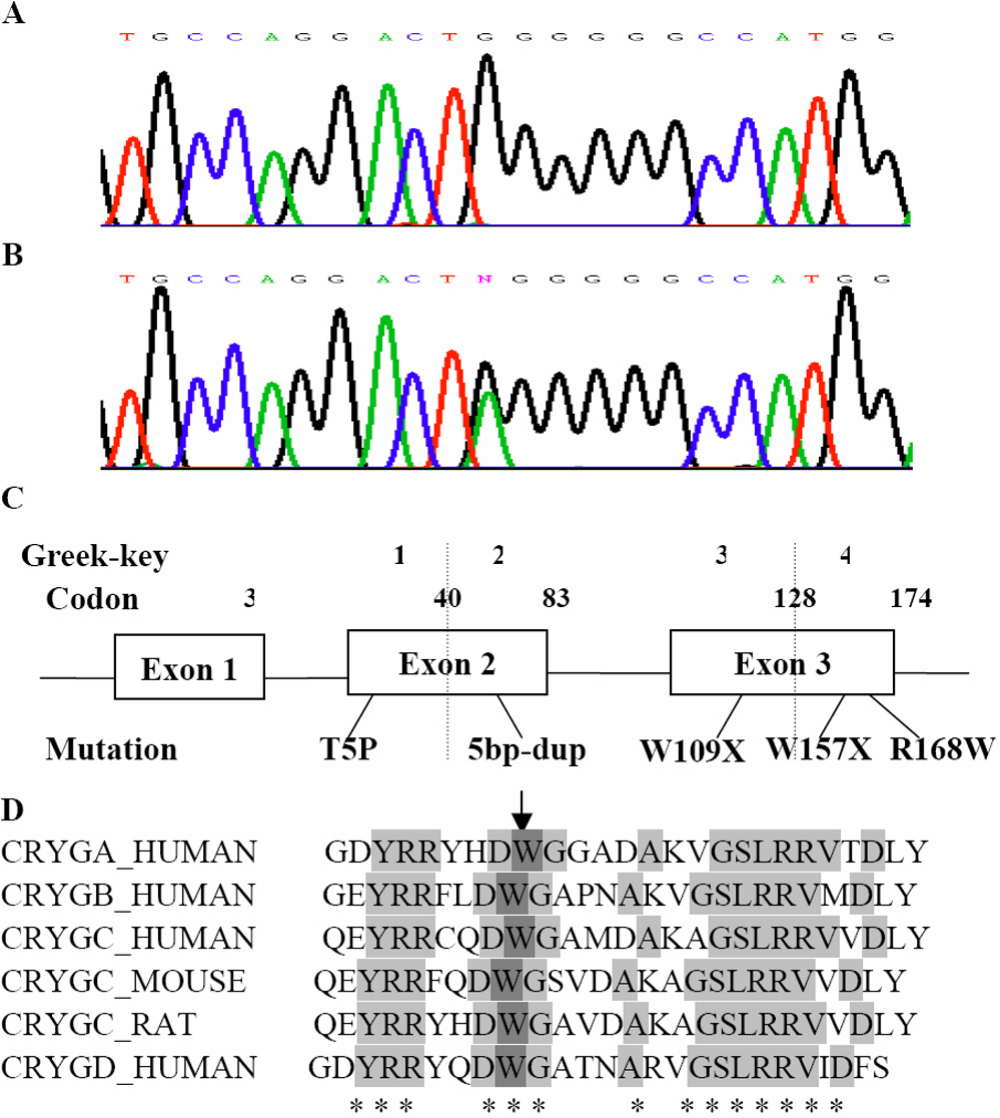
Mutational analysis of *CRYGC*. **A**: Sequence chromatograms of the wild type *CRYGC* allele show that the wild type gene encodes a tryptophan residue (TGG) at position 157. **B**: Sequence chromatograms of the mutant allele show a c.470G>A transversion that substituted a termination codon (TAG) for the tryptophan residue at position 157 (W157X). **C**: Exon organization and mutational profile of *CRYGC* are shown. Codons and the corresponding Greek key motifs are numbered above each exon. The relative locations of the W157X mutation and three other mutations associated with cataracts in humans are indicated. Mutations are numbered according to their amino acid position in the processed CRYGC protein. **D**: Multiple sequence alignments of *CRYGC* with the corresponding segments in human, mouse, and rat is exhibited together with human *CRYGA*, *CRYGB*, and *CRYGD* (sequences found using ClustalW2). The arrow indicates the W157X mutated position.

Sequence analysis of *CRYGC* identified a transversion in exon 3. This single nucleotide change was predicted to result in a c.470G>A nonsense or chain-termination mutation at codon 157, which changed a phylogenetically conserved tryptophan residue to a stop codon (W157X). The cosegregation of the c.470G>A transition in only affected members of the pedigree and its absence in 100 normal control individuals strongly suggested that the W157X substitution was a causative mutation rather than a benign single nucleotide polymorphism (SNP) in linkage disequilibrium with the cataract (Figure 3).

## Discussion

The association of developmental cataract and microcornea (congenital cataract-microcornea syndrome, microcornea-cataract [CCMC], OMIM 116150) makes up a distinct phenotype within the group of autosomal dominant congenital cataracts. CCMC has been considered a rare phenotype. The frequency of CCMC may be underestimated because the focus is often on the sight-threatening cataract and corneal diameters may pass unnoticed. Hansen et al. [48] identified five novel mutations by screening 10 CCMC families in nine already known cataract genes. The numbers of known mutations in CCMC are, however, still limited, which hampers evaluation of whether the appearance of CCMC phenotypes depends on specific mutations or closely linked modifying elements.

Herein, we report the identification of a novel nonsense mutation (W157X) in processed *CRYGC* that cosegregated with autosomal dominant nuclear cataracts and microcornea in a four-generation Chinese family. In addition to the novel W157X mutation, there are four other mutations in *CRYGC* that have been associated with cataracts including Coppock-like cataracts (T5P) [19], variable zonular pulverulent cataracts (5 bp duplication) [20], lamellar cataracts (R168W) [21], nuclear cataracts (R168W) [22], and nuclear cataracts (C109X) [23]. Therefore, in a manner similar to the nuclear opacities associated with the W157X mutation, these other *CRYGC*-related opacities involve the nucleus of the lens. This clinical manifestation is in agreement with the function of *CRYGC,* which is abundantly expressed at an early developmental stage in elongating fiber cells. *CRYGC* is expressed primarily in the lens nucleus, which is consistent with the location of the opacity in the nucleus.

Crystallins constitute the major proteins of the vertebrate eye lens, and they maintain the transparency and refractive index of the lens. Because lens central fiber cells lose their nuclei during development, these crystallins are made and then retained throughout life, requiring them to be extremely stable proteins. Mammalian lens crystallins are divided into α-, β-, and γ-crystallin families. β- and γ-crystallins are also considered a superfamily. Seven protein regions exist in β-crystallins: four Greek key motifs, a connecting peptide, NH_2_-terminal extensions, and COOH-terminal extensions. γ-Crystallins are a homogeneous group of highly symmetric, monomeric proteins. They are differentially regulated after early development. Four γ-crystallin genes (*CRYGA*, *CRYGB*, *CRYGC*, and *CRYGD*) and two pseudogenes (*CRYGE* and *CRYGF*) are tandemly organized in a genomic segment as a gene cluster.

Based on the crystal structure of human *CRYGC*, the W157X mutation lies in the COOH-terminal domain at the end of the fourth Greek key motif (Figure 3C). However, the deleterious effects of the W157X mutation on *CRYGC* folding, stability, and solubility are unclear. Interestingly, the T5P mutation, which is associated with Coppock-like cataracts, not only causes changes in conformation (partial unfolding) but also reduces protein solubility and stability [49]. Furthermore, the T5P mutation decreases interactions with wild type γC-crystallin and with αA-, αB-, and βB2-crystallins [50]. How the 5 bp duplication and R168W mutations, which are associated with variable zonular pulverulent cataracts and lamellar cataracts, respectively, exert their unique effects remains unknown. Further biophysical studies of these mutant proteins will be required to provide insights regarding the molecular pathology of *CRYGC*-associated cataracts.

Alignment of the CRYGC protein sequence from three different species with three human γ-crystallins revealed that the tryptophan residue at position 157 is highly conserved (Figure 3D). To further examine the structural change induced by the W157X mutation, we used the three-dimensional structure of γ-crystallin, which is characterized by the presence of four Greek key motifs. Motifs 1 and 2 are located in the NH_2_-terminal domain, and motifs 3 and 4 are located in the COOH-terminal domain [51,52]. The W157X mutation results in an in-frame stop codon at nucleotide 470 that leads to the truncation of 17 amino acids from the COOH-terminus of γC-crystallin. The corresponding alteration affects not only the length of the COOH-terminal arms but also the formation of the fourth Greek-key motif in γC-crystallin, which in turn disrupts the highly symmetric structure of γC-crystallin. The W157X mutation affects the Greek key motifs, and it is predicted to change the folding properties of γC-crystallin and/or affect its interactions with other crystallins. Furthermore, the resulting changes in the three-dimensional structure may ultimately lead to a decrease in the stability and/or solubility of γC-crystallin.

It is noteworthy that an autosomal dominant nuclear and radial cataract caused by a 6 bp deletion in the *CRYGC* has also been reported in the mouse [53]. A 6-bp deletion in exon 3 of the mouse γC-crystallin encoding gene (*Crygc*) is causative for the cataract phenotype. The mutation is therefore designated *CrygcChl3*. The deletion of bases 420−425 leads to a loss of two amino acid residues, Gly and Arg, in the fourth Greek key motif.

In summary, the present study has identified a novel nonsense W157X mutation in *CRYGC* associated with autosomal dominant cataracts and microcornea in a four-generation Chinese family. The association between microcornea and congenital cataracts in all affected individuals indicate that mutations disrupting lens biochemistry and physiology early in development can result in microphakia and subsequent microcornea as a secondary effect of damage to the lens.
